# Gold *vs*. Amalgam

**Published:** 1883-04

**Authors:** U. Smith


### GOLD vs. AMALGAM.

Operative dentistry is now divided into two factions, "Conservative" and "New Departure," each party in every journal, convention, association, and by private office intercourse, is putting forth its respective merits. These agitations have now been continued a length of time until the innocent public are, to a great extent, converts to one or the other theory. The Conservative faction maintain that gold is the only *par excellence* material for a safe and permanent preserver of carious teeth ; while the other faction as tenaciously maintain that plastic materials, especially tin and silver alloys, rival gold as universal filling. Able practitioners, of long experience, arrive at different conclusions. So it has been and ever will be in every department of science. Each mind is a world unto itself ; therefore it generates theories peculiar to its own reasoning, and molds a corresponding manifestation. With the same means at command, one person, with a reputation for common sense and years of practice, becomes only a medium mechanic, while another, in the same situation, surprises the world with his productions. One surgeon will declare, upon honest convictions, that the operation cannot be performed without destroying the patient's life, while another, of less experience but better judgment, declares to the contrary, and is successful. Napoleon's campaigns, under his supervision, were successful, when, if guided by one of his selected marshals, would have been disastrous.

A. T. Stewart, alone in his office, planned and guided to successful consummation, commercial enterprises over combined competitors. A hundred commercial geniuses united were beneath his level.

After these desultory observations, let us consider the respective merits of the two factions that stand arrayed as opposing forces in the science of preserving the natural teeth.

First—Is not gold the purest and most ductile of all the known metals? Most assuredly. How do we know this? Because its resistance to oxidation is greater; in fact, acids that soon oxidize silver, or silver and mercury combined, are not perceptible when applied to gold. Are not oxides of many metals poisons? Most assuredly. Then, being poisons, are they not deleterious to the secretions; deadly destructive to vitality of osseous tissue? Assuredly. This being the case, should they not be avoided, if not altogether, as much as possible?. It seems to me that an affirmative answer would be correct; but the New Departure fraternity assert that amalgam preserves teeth when all other fillings, especially gold, fail, and therefore lose sight of everything else. They have tried both, therefore they know all about it. People are carrying tons of amalgam in their teeth and still live. That settles it. They further argue that gold fillings, solidified as much as the mallet and condensers will admit, and polished to mirror brightness, soon leak, decay sets in, fillings loosen, drop out,—patient returns within a year after the operation, with lump of gold, saying, "Doctor, that fine filling has come out; here is;" find tooth very much decayed,—excavate anew, fill with amalgam; no more leakage, stays well five or ten years. This proves that gold fillings, however well compacted, are "trash." Then the New Departure cock flaps his wings and crows long and loud, until the whole country hears him, from Maine to Georgia. Now, why did his gold filling leak? for assuredly he condensed it enough. Was it packed closely againt the walls of the cavity? Apparently it was, though in reality it was *not*, for if it had been well packed against the cavity walls throughout, no leakage could be possible. "Aye," says the apostle of New Departure, "that is the very difficulty,—so rarely can it be done." Well, let us see: You have made, say two or more retaining cuts in your cavity, forced your gold into these undercuts, then continued filling with condenser of the usual size, until the cavity is filled and contoured, and as much as the occasion requires.

The filling, though very *nice*, is a failure, merely because you have condensers three times too large ; therefore the filling is diverted, in each layer, to the center,—in fact bailed up. It does not hug the sides of the cavity only in places, consequently moisture works in, caries goes on, and shortly the *splendid* gold plug drops out. I know how it is from experience. My fillings have dropped out lots of times, to my chagrin, and to the annoyance of my patients. I did not abandon gold, but looked for the cause of failure. After ascertaining the cause, I set out on a new departure principle ; that is, I threw aside the coarse condensers usually advertised, substituted them with fine points such as are generally used for root fillings, bent them into the required shapes, took gold foil, Nos. 4 and 5, cut each leaf into three or four slips, rolled each slip loosely, then cut them into pieces of different sizes ; after heating them to redness, I condense one piece in place, holding it there firmly, adding on piece after piece, closely condensing against the sides of cavity, which only fine points can do, then when sufficient gold is in place, condense as usual, pressing firmly one side with one hand, while malleting the other, this secures against any possible vibration. The hand mallet is generally preferable to the automatic, especially for frail teeth.

My experience convinces me that this system, if well followed, will be effectual against leakage, or danger of fillings giving way. Screws are great auxiliaries where there is a lack of base or want of support. Dentists should avoid cutting away healthy dentine, and use screws in lieu thereof. For my part I am disgusted with this New Departure literature. I hope the genius of Conservative dentistry will choke it out, as tares from among wheat. Plastic fillings are useful, in fact indispensable auxiliaries ; but to give them precedence over gold, is to declare the moon's cold magnetic rays more conducive to health and vigor than the warm electric rays of the sun.—*Dr. U. Smith, in the Ohio State Journal.*

